# Fenestrated Basilic Vein Traversed by a Rare Variant of the Medial Brachial Cutaneous Nerve

**DOI:** 10.7759/cureus.94802

**Published:** 2025-10-17

**Authors:** George Triantafyllou, George Tsakotos, Alexandros Samolis, Theodore Troupis, Maria Piagkou

**Affiliations:** 1 Department of Anatomy, School of Medicine, Faculty of Health Sciences, National and Kapodistrian University of Athens, Athens, GRC

**Keywords:** anatomy, basilic vein, cadaveric dissection, medial cutaneous brachial nerve, variation

## Abstract

Variations of the upper limb’s neurovascular structures hold significant clinical relevance for surgical, anesthetic, and vascular procedures. Although variations in the formation and branching of the brachial plexus and venous architecture of the arm are common, neurovascular crossings involving venous fenestrations are exceptionally rare. During a routine cadaveric dissection of a 68-year-old male, a fenestrated basilic vein (BV) was identified on the right arm, approximately 12.7 mm distal to the inferior border of the pectoralis minor muscle. The fenestration measured 17.6 mm in length and consisted of distinct medial and lateral limbs. One brachial vein drained into the lateral limb, while the other joined the BV distal to the fenestration. At the same level, the medial brachial cutaneous nerve (MBCN) was formed by its typical medial root and a lateral variant branch communicating with the ulnar nerve (UN). Notably, this communicating branch traversed the fenestrated segment of the BV. The contralateral arm did not have any neurovascular variation. This rare configuration likely results from incomplete condensation of the primitive venous plexus combined with aberrant axonal guidance during brachial plexus development. Such variants are of clinical importance due to their potential impact on venous catheterization, axillary block anesthesia, and vascular access surgery.

## Introduction

The neurovascular anatomy of the upper limb exhibits both typical and atypical configurations, which are of great significance in surgical and clinical practice. The topographical relationships among these structures are intricate and often display considerable variation.

The brachial plexus (BP), which provides motor and sensory innervation to the upper limb, typically arises from the anterior rami of the C5 to T1 spinal nerves. It gives rise to several terminal branches, including the median nerve (MN), ulnar nerve (UN), radial nerve (RN), musculocutaneous nerve, subscapular nerve, thoracodorsal nerve, and medial brachial cutaneous nerve (MBCN) [1]. BP exhibits substantial morphological variability in both its formation and branching patterns [1]. Three recent evidence-based meta-analyses have collectively summarized these variants, addressing the formation of the BP, branching of its supraclavicular part, and branching of its infraclavicular part [1-3].

The MBCN is a purely sensory branch derived from the medial cord of the BP, carrying fibers from the C8 and T1 spinal nerves. The nerve courses medial to the axillary vein (AV), accompanying it through the axilla and arm. Along its course, it provides cutaneous branches to the skin over the medial aspect of the arm and often communicates with the intercostobrachial nerve in the upper arm. Near the mid-arm, it pierces the deep fascia, terminating as small branches supplying the skin of the distal medial arm and proximal forearm [4].

Similarly, the venous system of the upper limb shows high morphological variability. Typically, the basilic vein (BV) drains blood from the medial surface of the forearm and arm into the AV. In contrast, the cephalic vein drains the lateral aspect [5]. In addition, two brachial veins accompany the brachial artery and usually converge into the BV. These venous patterns are notably variable and have important implications for surgical interventions and catheterization procedures [6].

Previous studies have reported several cases of nerves traversing venous fenestrations in different anatomical regions. Examples include the auriculotemporal nerve passing through the superficial temporal vein, the accessory nerve penetrating the internal jugular vein, and the facial nerve crossing the retromandibular vein [7-9]. In this context, the present report documents a rare case of a nerve penetrating a venous fenestration in the upper limb.

## Case presentation

Upper limb dissection of a 68-year-old male cadaver was performed. The body was derived from the “Body Donation Program” of the responsible authorities (affiliation 1) [10]. Skin, subcutaneous fat, and superficial fascia of the upper limb were dissected. The limbs were free of any physical deformity or trauma.

On the right side, an atypical neurovascular configuration was recorded. Distally to the inferior border of the pectoralis minor muscle (12.7 mm), a fenestrated BV was recorded. The fenestration was 17.6 mm long, with a lateral and medial limb. One of the brachial veins was drained into the lateral limb of the fenestration, while the second one into the BV distally to the fenestration.

**Figure 1:**
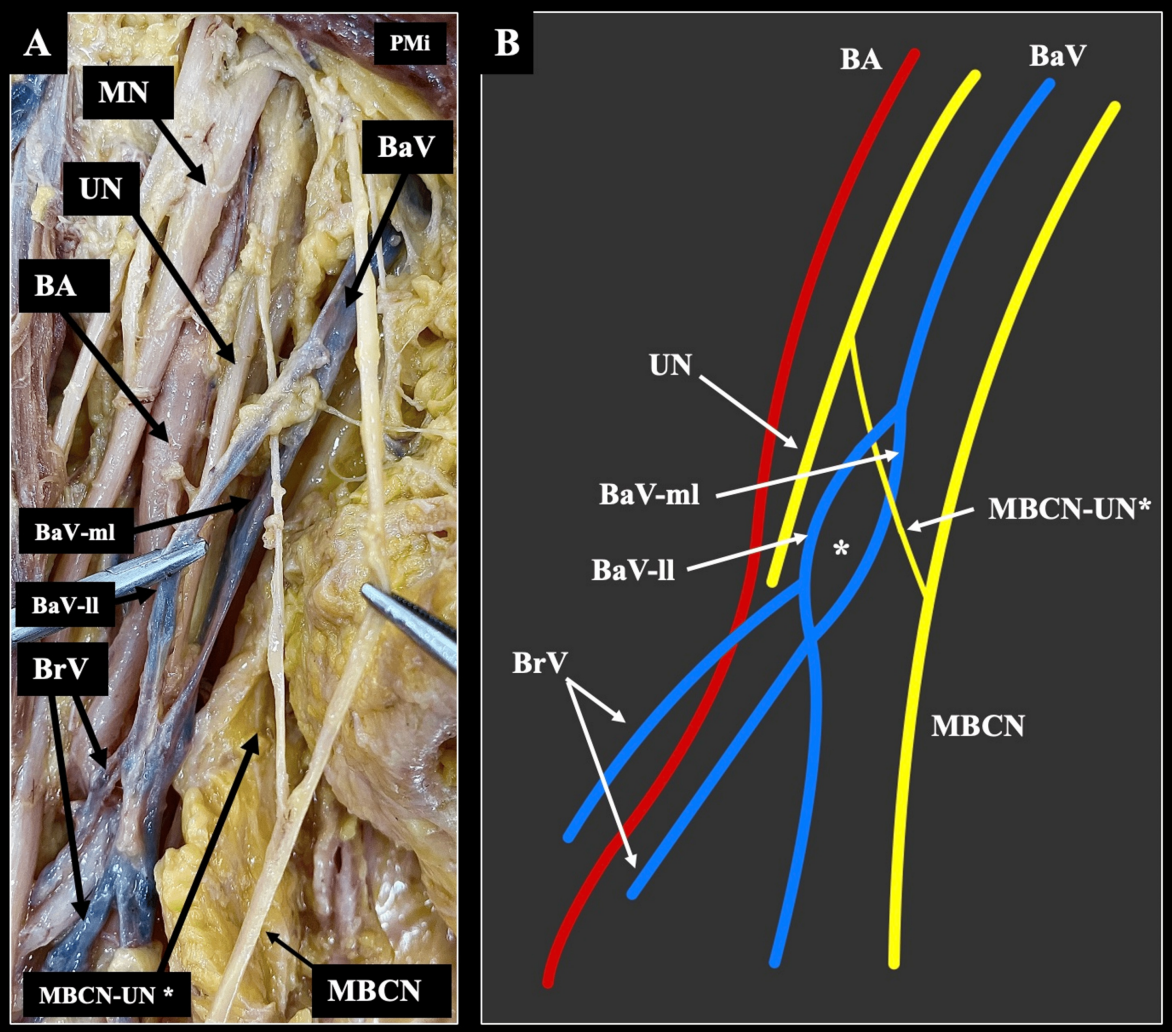
A variant interconnection of the medial brachial cutaneous nerve traverses the fenestrated basilic vein. (A) Dissection of the right upper limb of a 68-year-old male cadaver demonstrates the fenestrated basilic vein (BaV) – with a lateral limb (BaV-ll) and a medial (BaV-ml) - penetrated by the interconnection of the medial brachial cutaneous nerve (MBCN), with the ulnar nerve (UN) defined as MBCN-UN*. The brachial vein (BrV), brachial artery (BA), and median nerve (MN) are shown in their usual topography. (B) Schematic illustration of the same region, showing the transvenous course (*) of the MBCN-UN* through the fenestrated segment of the BaV and its relationship with surrounding structures. PMi: pectoralis minor muscle

At the same level, the terminal branches of the BP were typically identified. However, we observed a peculiar variant. The MBCN was typically formatted by its typical origin from the medial cord, but received laterally, a variant branch that was an interconnection between the UN and the MBCN. This branch was coursing through the fenestrated segment of the BV (Figure 1). Regarding the origin, course, and distribution, the neurovascular elements did not have any variation. On the left side, the neurovascular anatomy was typically identified.

## Discussion

Herein, we described the interconnection between the UN and the MBCN that was coursed through a fenestrated BV. A few cases have documented analogous neurovascular variations involving the medial cutaneous nerves and nearby veins or nerves of the upper limb. Roy and Sharma described a case where the medial cutaneous nerve of the forearm perforated the AV, forming a tunnel-like structure [11]. This configuration was proposed to arise from aberrant axonal entrapment during embryonic vascular development [11]. A more recent case by Raviteja et al. reported the MBCN piercing the AV without forming a tunnel or communication with the venous lumen [12]. Unlike Roy and Sharma’s case, this variation involved a simple transvenous passage and included a communication with the intercostobrachial nerve [11-12].

The MBCN interconnections have been infrequently reported in the current literature. In a cadaveric study, Piagkou et al. reported intercostobrachial nerves’ interconnections with the MBCN in 5% of cases [13]. Moreover, Marathe et al. [14] observed an unusual neural link between the MBCN and the RN within the axilla, crossing the third part of the axillary artery and lying in proximity to the AV. Developmentally, such communications may arise from persistent embryonic connections or aberrant axonal guidance, influenced by chemotactic gradients during limb innervation [13,14]. Functionally, these interconnections may underlie the variable sensory overlap and cross-innervation patterns observed clinically in the medial arm and forearm, underscoring their significance in surgical and anesthetic procedures involving the axilla and upper limb [13,14].

The BV demonstrates extensive morphological variability in its course, confluence, and relationships with the brachial and AV. Anaya-Ayala et al. classified BV-brachial junctions into three main types based on duplex ultrasound mapping of 426 upper limbs: the typical type 1 pattern (66%) where the BV joins paired brachial veins in the proximal third of the arm; type 2 (17%) with a distal confluence but persistent paired brachial veins; and type 3 (17%) where a single unpaired brachial vein joins the BV in the mid- or lower arm [5]. The latter types were considered clinically significant due to their impact on vascular access creation and risk of inadvertent inclusion of deep veins during BV transposition [5]. Complementing these findings, Yang et al. described further venous complexity within the axillary region, identifying duplication of the AV in 17.5% of limbs and the presence of venous loops through which the MBCN occasionally passed (7.5%) [6]. The authors attributed these configurations to incomplete condensation of the embryonic venous plexus and noted their relevance in catheterization and axillary surgeries [6]. In comparison, the current case represents a distinct variant where the BV displayed a fenestration distal to the pectoralis minor. Unlike the duplications or distal junctions of the AV described in prior reports, this fenestration created a transvenous corridor through which an interconnecting branch between the UN and the MBCN coursed.

The concurrent variants described in the current case can be explained by deviations during the intricate development of the upper limb’s neurovascular patterning. During the fifth week of embryogenesis, the venous drainage of the limb bud arises from the superficial and deep venous plexuses, with the postaxial channel forming the BV and the axial vein later giving rise to the brachial veins and AV [6]. Failure of complete condensation or partial persistence of this primitive plexus may result in venous duplications or fenestrations, as documented by Yang et al., who observed duplicated axillary veins in 17.5% of cases and proposed incomplete venous coalescence as the developmental mechanism [6]. Parallel to venous remodeling, motor and sensory axons of the BP extend into the limb [1,14]. Perturbations in these molecular gradients or in local vascular topography may redirect axonal growth, resulting in atypical interconnections like those between the MBCN and UN or RN described in the current and previous reports [13,14]. Clinically, these neurovascular anomalies bear clinical implications. A fenestrated BV may pose challenges during venous catheterization, axillary block anesthesia, and brachio-basilic arteriovenous fistula creation, with risks of inadvertent puncture or inadequate venous drainage [5,6]. Moreover, the nerve traversing the venous fenestration, as in the present case, may be vulnerable to entrapment or compression within the venous lumen - especially under conditions of venous distension or limb hyperabduction - potentially leading to paresthesia or sensory deficit along the medial arm. Similar venous-neural crossings in the axilla have been linked to sensory disturbances and thrombosis due to venous stasis [6,11].

## Conclusions

The present case highlights a unique neurovascular variation characterized by a fenestrated BV traversed by an interconnecting branch between the UN and the MBCN. This configuration expands the known spectrum of venous and neural anomalies of the upper limb. Embryologically, it likely results from incomplete condensation of the primitive venous plexus coupled with aberrant axonal guidance during BP development. Clinically, recognition of such variants is essential during axillary surgeries, venous catheterization, vascular access creation, and regional anesthesia, as unawareness may lead to inadvertent vascular injury or sensory deficits. Therefore, careful intraoperative dissection is recommended to prevent iatrogenic complications arising from rare neurovascular configurations such as the one described here.
